# Experiences of being diagnosed with unruptured intracranial aneurysm in a population-based health survey: A qualitative interview study

**DOI:** 10.1016/j.bas.2026.107469

**Published:** 2026-10-09

**Authors:** Mathilde Vea Iversen, Ingvild Mathiesen Rosenlund, Jørgen Gjernes Isaksen, Alexandra Brandt Ryborg Jønsson, Tor Ingebrigtsen

**Affiliations:** a Department of Clinical Medicine, UiT the Arctic University of Norway, Hansine Hansens veg 18, Tromsø, 9019, Norway; b Department of Neurosurgery, University Hospital of North Norway, Hansine Hansens veg 67, Tromsø, 9019, Norway; c Department of Community Medicine, UiT the Arctic University of Norway, Hansine Hansens veg 18, Tromsø, 9019, Norway; d Department of People and Technology, Roskilde University, Universitetsvej 1, Roskilde, 4000, Denmark

**Keywords:** Unruptured intracranial aneurysms, Screening, Incidental findings, Quality of life, Psychological outcomes

## Abstract

**Introduction:**

Most unruptured intracranial aneurysms (UIA) are small and carry a low rupture risk. Many patients are therefore not treated with occlusion but managed conservatively. Evidence on the psychological impact of living with an untreated UIA is limited and conflicting.

**Research question:**

To explore experiences of receiving a diagnosis of a UIA and having to live with it over time.

**Material and methods:**

The Tromsø7 health survey examined 1862 participants with magnetic resonance angiography and 85 diagnosed with conservatively managed UIAs were eligible for inclusion. We invited 24 based on purposive maximum-variation sampling according to age, sex, and aneurysm size, and 18 consented. Twelve participated in semi-structured interviews before data saturation was achieved. We excluded two because the interviews revealed exclusion criteria (cognitive impairment and treated aneurysm). Data from 10 interviews conducted median eight years after diagnosis were included in a thematic analysis.

**Results:**

In retrospective narratives, many participants recalled experiencing serious worry or fear shortly after the diagnosis. Most described a subsequent process of adaption to the uncertainty through trust in clinicians, resilience, normalization and meaning-making, which enabled them to reestablish normality in everyday life. Concerns could resurface around follow-ups. Few described persistent anxiety at the time of the interview. The diagnosis motivated healthy lifestyle changes for many.

**Discussion and conclusion:**

The interviews revealed substantial psychosocial impacts of having to live with untreated UIAs. We suggest these insights could inform decision-making in contexts where clinicians and patients must balance the potential benefits and burdens of imaging and surveillance.

## Highlights

• Quantitative studies suggest that incidental detection of an UIA is well-tolerated.
• Qualitative studies indicate that quantitative methods may not capture all experiences.
• This interview-study found substantial initial worry which resolved over time.
• Follow-up examinations triggered recurrence of concern.

## 1 Introduction

### 1.1 Background

Unruptured intracranial aneurysms (UIA) are common with a prevalence of 1.9-7.4% in population-based studies (Müller et al., 2013; Li et al., 2013; Johnsen et al., 2022). The wide range reflects differences in use of imaging modalities and cut-offs for aneurysm size between studies. The highest prevalence was observed in a study using high-resolution 3 T magnetic resonance angiography (3T MRA) to identify aneurysms ≥1 mm (Johnsen et al., 2022).

Increased use of diagnostic imaging for other conditions and improved imaging quality have caused increasing detection rates for incidentally diagnosed UIAs (Gabriel et al., 2010; Smith-et al., 2019). Most such aneurysms are small with a low risk of rupture and subarachnoid haemorrhage (SAH) (Macdonald and Schweizer, 2017). In systematic reviews, the overall complication risk related to preventive treatment with surgical or endovascular occlusion range from 5 to 12%, depending on definitions and patients’ age (Algra et al., 2019; Sturiale et al., 2013). Consequently, many aneurysms are not treated with occlusion, but managed conservatively with risk factor modification including smoking cessation, antihypertensive medication and follow-up examinations (Thompson et al., 2015).

There is limited and partly conflicting evidence regarding the psychosocial impact of being diagnosed with a UIA and advised against treatment. Most previous studies on psychosocial outcomes used quantitative methods. A recent meta-analysis suggests a potential negative impact on anxiety and quality of life, although the findings were heterogeneous and appeared to be time-dependent, with reduced impact over time after diagnosis (Penchev et al., 2025).

Generic questionnaires have limited sensitivity in capturing experiences and psychosocial effects of screening and incidental findings (McCaffery and Barratt, 2004). A previous mixed-methods study suggested that living with a conservatively managed UIA may involve psychological distress and changes in everyday life that are not captured by quantitative measures (Jelen et al., 2023). The study used qualitative interviews after median 14 months, and reported that reactions such as anxiety, sadness and worry entailed lifestyle changes including e.g. avoidance of straining-intensive exercise and modification of work-related performance.

We have previously conducted a quantitative longitudinal study of participants diagnosed with UIA in the Tromsø Study, a population-based health survey. At a median of four years after diagnosis, participants with non-occluded UIAs generally did not differ from controls or from their pre-diagnosis levels on generic measures of quality of life, psychological distress, or health anxiety (Rosenlund et al., 2024). Whether these findings reflect an absence of psychosocial impact, or limitations in quantitative instruments, remains uncertain. This provided the unique opportunity of a long-term follow-up with an in-depth study of participants from the same population-based cohort to inform the knowledge gap about long-term psychosocial outcomes of living with a non-treated UIA. Therefore, this qualitative study explored the experiences of a subsample of participants diagnosed with UIA in the Tromsø study.

### 1.2 Aim

We aimed to explore participants’ experiences of receiving a diagnosis of a UIA and living with a conservatively managed aneurysm over time.

## 2 Methods

### 2.1 Qualitative approach and research paradigm

This is a cross-sectional qualitative study of participants who had UIAs detected in a population-based health survey. The study was informed by a phenomenological approach without use of a guiding theory. We used semi-structured individual interviews and thematic analysis, and report in accordance with the recommendations in the Standards for reporting qualitative research (O’Brien et al., 2014).

### 2.2 Researcher characteristics and reflexivity

The first author (MVI) conducted all interviews. MVI is a medical doctor and PhD student. She was not involved in the participants’ medical care, did not have access to their medical records and had no information about participants’ health except the diagnosis before the interviews. The multidisciplinary research team includes two neurosurgeons (JGI, TI), a psychiatrist in training (IMR) and an anthropologist (ABRJ). JGI counseled and followed up the participants at the neurosurgical outpatient clinic after the diagnosis. Eligible pseudonymized participants from the previous quantitative study were selected by IMR. Their identifiers were decoded by research administrative personnel in the Tromsø study, which provided MVI the list of identified eligible participants. MVI invited them to participate. At this stage, all participants were anonymized for the other members of the research team.

We departed from the negative findings in our previous quantitative study (Rosenlund et al., 2024), were influenced by contrary findings in a previous qualitative study (Jelen et al., 2023) and strived not to hold specific assumptions. The multidisciplinary composition of the research team contributed to reflexive discussions during data analysis.

### 2.3 Context

The Tromsø Study is a population-based repeated cross-sectional health survey (Jacobsen et al., 2012). The seventh iteration (Tromsø7) was conducted in 2015-2016. All inhabitants ≥40 years (n = 32 591) in Tromsø municipality were invited and 65% (n = 21 083) consented. A subset (n = 1862) of randomly selected participants were examined with 3T MRA of the brain (Johnsen et al., 2022).

Fig. 1 shows that 143 (8.5%) were diagnosed with UIAs. We excluded 21 participants who had aneurysms with extradural location because such aneurysms cannot cause SAH. Another 21 were excluded because they were not informed about the finding based on an assessment that communicating the diagnosis would not benefit them (Johnsen et al., 2022). The assessments were done discretionary by neurosurgeons and neuroradiological interventionists in multidisciplinary vascular team meetings according to pre-specified criteria (small aneurysms with ignorable rupture risk in participants with advanced age and/or significant comorbidity). The remaining (n = 101) attended the neurosurgery outpatient clinic at the University Hospital of North Norway (UNN). Five underwent preventive treatment with occlusion after individual assessment, including PHASES (population-, hypertension-, age-, size-, earlier SAH-, site-) scoring (Greving et al., 2014). The other 96 received diagnosis information, advice on smoking cessation and blood pressure control when appropriate, and follow-up including repeated imaging (Rosenlund et al., 2024).

**Fig. 1:**
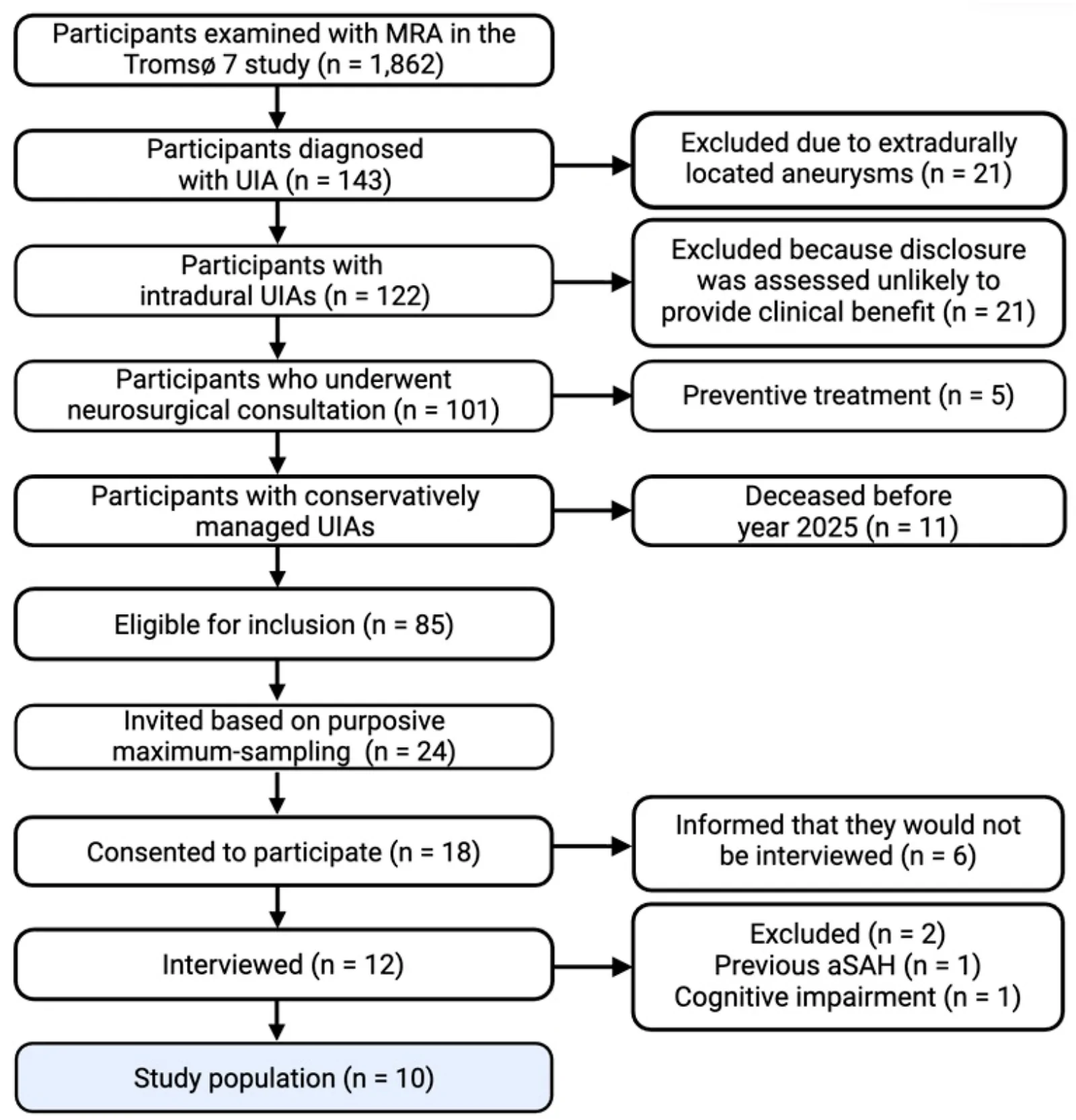
Flowchart displaying the process for recruitment of participants.

### 2.4 Sampling strategy and units of study

We received permission from the Tromsø Study to mail an invitation letter and a consent form to participants from Tromsø7. 85 of the 96 participants with conservatively managed UIAs were alive and thus eligible for inclusion (Fig. 1).

We used purposive maximum-variation sampling according to age, sex and aneurysm size. Sex was selected to ensure gender equality, while aneurysm size and age are key variables according to PHASES because the rupture risk increases with size and the complication risk related to treatment with age (Greving et al., 2014).

We anticipated that ±15 participants would be required to reach data saturation, expected 80% response rate and invited 24 to enable a maximum sample size near 20. 18/24 (75%) individuals invited consented and 12 were interviewed (Fig. 1). Coding and analyses were done concurrently. The interviewer recorded no new codes or themes after interview no. 9 and presented a temporary analysis for the research team. We collectively assessed the data as sufficient to address the research questions and considered saturation to have been reached. The recruitment was stopped, and the remaining six individuals who had consented were informed that they would not be interviewed.

Exclusion criteria were cognitive impairment, previous aneurysm occlusion, language other than Norwegian or English, and a history of SAH. One participant was excluded because the interview revealed that the participant had undergone treatment after SAH. One more was excluded after discussion in the research team because the interviewer considered the participant to have cognitive impairment to such a degree that no meaningful information was provided. The final sample therefore comprised 10 participants (Malterud et al., 2016). Table 1 shows the participants’ characteristics.

**Table 1:** List of participants included in the analysis.

Sex	Age at diagnosis (years)	Age at interview (years)	Years since diagnosis	Aneurysm size (mm)
Woman	48	56	8	3
Woman	51	59	8	2
Woman	58	66	8	3
Woman	63	71	8	5
Man	51	60	9	4
Man	54	61	7	2
Man	60	65	5	3
Man	65	73	8	6
Man	68	77	9	7
Man	75	83	8	5

### 2.5 Ethical issues pertaining to human subjects

The study was approved by the Regional Committee for Medical and Health Research Ethics North (record number 126174/2020) and the Data Protection Officer at UNN (record number 02609/2021). All participants provided written informed consent. They were informed that their responses would remain confidential, be anonymized to the neurosurgeons in the research group and other clinicians, and that participation would have no implications for current or future care.

### 2.6 Data collection methods

The interviews were conducted between 27 March 2025 and 12 June 2025, a median of 8 (range 5-9) years after the diagnosis. All were conducted face-to-face in Norwegian at the UNN’s neurosurgical outpatient clinic and lasted 60-100 min. They were audio-recorded and transcribed verbatim by MVI. The second author (IMR) attended the first two interviews in a mentoring role (implying she was not blinded for these two participants identity).

### 2.7 Data collection instruments and technologies

The interview guide (see supplementary file 1) was developed by MVI and subsequently reviewed and revised in collaboration with the co-authors. It was pretested with colleagues and non-expert acquaintances and refined to improve clarity and suitability. We used open-ended questions to avoid restricted responses. The questions addressed participants’ recollection of experiences and daily living before and after diagnosis, how this developed over time, and current well-being. One question regarding concerns about familial risk was removed after the first two interviews because we considered it potentially anxiety-provoking and not essential to the study aims. The topic nevertheless emerged spontaneously in all subsequent interviews. We retained all responses and analyzed them on equal terms.

### 2.8 Data processing

The transcripts were anonymized and stored on an encrypted institutional server with restricted access. The data were manually coded in Norwegian and organized in Microsoft Word, using color-coding, margin comments, and hierarchical text structuring. An audit trail documenting coding decisions and revisions was maintained throughout the process. MVI translated the codes and quotations to English.

### 2.9 Data analysis

MVI did the primary analysis of the interview data under mentoring by ABRJ and according to Braun and Clarke’s framework for thematic analysis (Braun and Clarke, 2006; Clarke, 2016). The coding was inductive, with codes developed from the data and not from a predefined guiding theory. The transcriptions were thoroughly reviewed to identify initial themes and recurrent meaning. Similar codes were grouped into subcategories, from which broader themes, sub-themes and quotations relevant to inform the research question were developed.

Next, MVI presented the themes and illustrative quotations for the research team and received feedback about ambiguities, overlapping across themes, inconsistencies and coherence between interpretations and quotations. Based on this feedback, MVI revisited transcriptions and codes to refine the themes and the selection of quotations. This was done iteratively (three times) until the team considered the analysis complete.

All participants contributed to all codes identified in the final analysis, and quotations were retrieved from seven participants. Supplementary Table 1 shows the distribution of participants’ quotations per theme.

### 2.10 Techniques to enhance trustworthiness

Throughout the analysis, we reflected critically on how our preconceptions and potential biases could influence the analysis. In the iterative discussions about themes and selection of quotations, we used investigator triangulation which included clinical neurosurgical (JGI, TI), psychological (IMR) and anthropological (ABRJ) perspectives to counteract interpretation- and confirmation-bias. The authors who had not been involved with the prior quantitative study (MVI and ABRJ) were encouraged to challenge inputs possibly influenced by such bias.

## 3 Results

The study captured participants’ retrospective narratives of experiences after receiving the diagnosis and identified three main themes: “Receiving and making sense of knowledge causing uncertainty”, “maintaining normality and identity” and “translating the diagnosis into action and acceptance”. The themes were divided into subthemes as illustrated in Fig. 2.

**Fig. 2:**
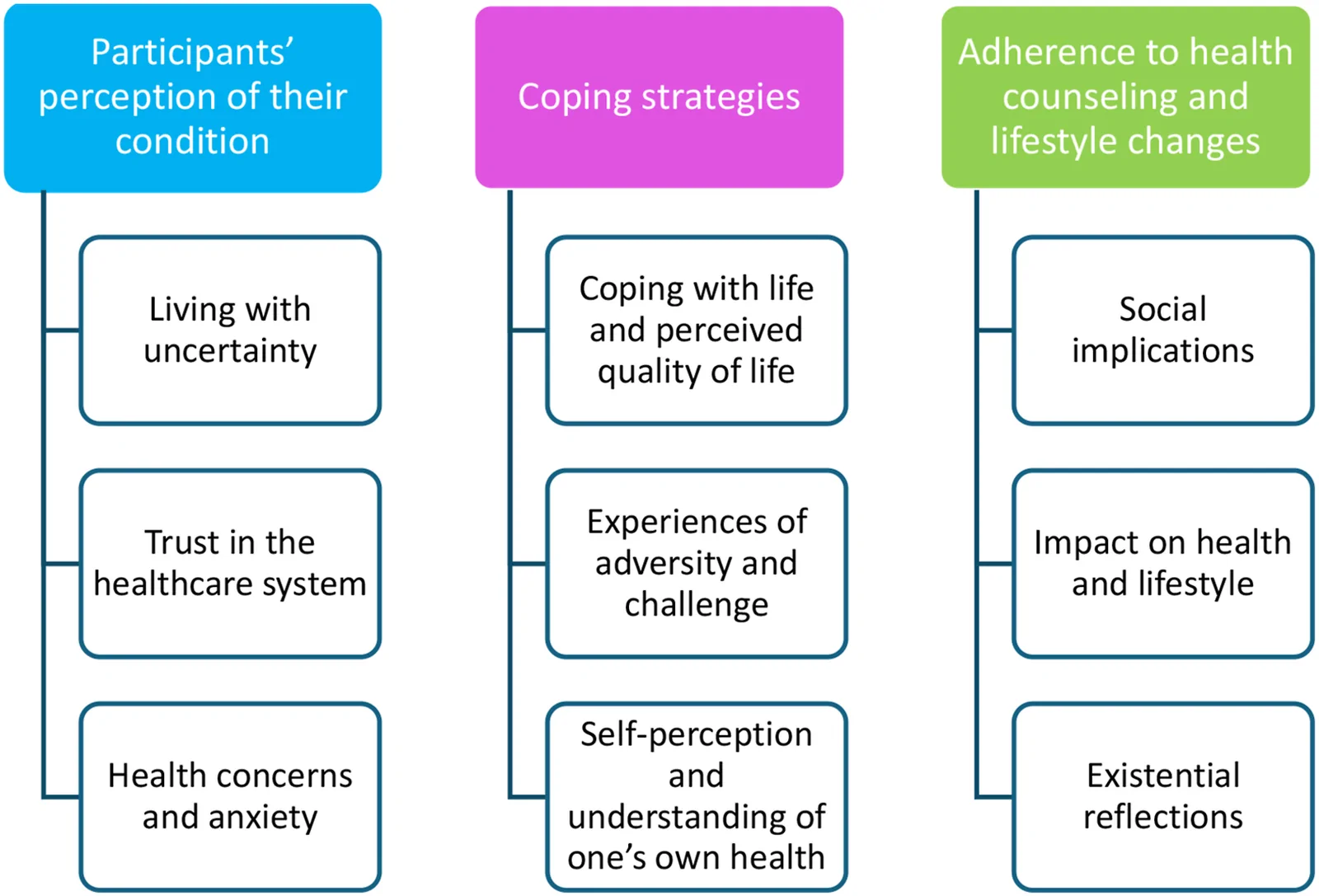
Display of themes and subthemes identified by the thematic analysis.

Many participants expressed how they had felt concern and uncertainty at the time of diagnosis but recalled that they carried on with their lives as normal after receiving thorough information about the aneurysm. Some reported that they had felt reassurance in receiving follow-up, while others wished they never knew about the diagnosis. Some had forgotten about their UIA when they were invited to participate in the present study.

### 3.1 Receiving and making sense of knowledge causing uncertainty

#### 3.1.1 Living with uncertainty

Many participants recalled that being diagnosed with an asymptomatic yet potentially serious condition introduced uncertainty into their everyday life. This was described as an inherent part of the diagnosis, particularly because the aneurysm was invisible and impossible to self-monitor:

*“In the head, you cannot see anything. (…) Since it is something that cannot be seen, one has no control over it.”* (Participant no. 1).

Several participants described an initial period of fear, especially before early follow-up, followed by a gradual reduction in worry as stability was confirmed over time:

*“I was afraid - terrified, in fact - before the first examination. (…) Actually, it wasn’t until after the final follow-up, three to four years later - when it hadn’t grown at all - that I was able to put it completely out of my mind.”* (Participant no. 5).

However, uncertainty could resurface when follow-up was discontinued or when participants felt they lacked updated information. Clear information and regular follow-up were described as key factors to feel secure. Without these, some anticipated that worry would have been more intrusive and tied to bodily sensations:

*“If I hadn’t had the regular check-ups, I think I would have spent more time worrying (…). I would have been convinced that the pain I feel on this side of my head was related to the aneurysm”* (Participant no. 6).

Participants actively negotiated uncertainty, balancing awareness of potential risk with strategies that allowed them to maintain everyday functioning. Temporal distancing was also used as a strategy to manage uncertainty. By framing potential adverse events as hypothetical or distant, they were able to preserve a sense of stability:

*“If you start thinking about what would happen if it ruptured, then it’s a different story.”* (Participant no. 9).

#### 3.1.2 Trust, information and surveillance

Participants recalled that experiences were strongly shaped by their interactions with healthcare professionals and by their confidence in medical expertise. Trust in medical expertise influenced how they interpreted risk, understood the diagnosis, and felt reassured in the absence of occlusion of the aneurysm.

A dominant pattern was that trust stabilized, enabling participants to manage uncertainty by relying on clinicians’ expertise and judgment. When healthcare professionals communicated confidence and provided clear explanations, participants often relied on this:

*“The doctor explained that many people are born with this condition (…). Therefore, there is no reason to worry, because we simply do not know whether anything is wrong. This was reassuring to me.”* (Participant no 5).

Conversely, ambiguous explanations, limited follow-up or inconsistent information could reintroduce doubt and prompt a renewed need for reassurance. In these moments, participants questioned whether the assessment was sufficient or whether important information might have been overlooked:

*“I then consulted a doctor who reassured me, saying, “Yes, it’s fine. It isn’t growing, nothing to worry about*.*” I felt a bit skeptical, however, since it was only a single examination.”* (Participant no. 1).

Clear personalized communication emerged as essential for sustaining trust. Participants emphasized that verbal dialogue, where risks, limits, and recommended actions were explained in accessible terms, was far more reassuring than written information, which was often perceived as vague or overly general. Trust also appeared as a dynamic and ongoing negotiation, shaped by continuity of care and evolving medical recommendations. When follow-up did not align with participants’ expectations, uncertainty could resurface, highlighting the need for predictable structures and repeated confirmation. Shifts in clinical guidelines or imaging practices were interpreted differently: some viewed them as medical progress, while others as unsettling, underscoring the provisional nature of medical knowledge.

Despite these variations, several participants expressed strong confidence in their clinicians and adhered closely to medical advice, illustrating how trust can anchor coping and guide health-related behavior:

*“I always take my medications as prescribed by my doctor (…). It has been recommended by the neurosurgeons as well that I need to reduce my blood pressure. It is a very important priority - to bring it down and keep it under control.”* (Participant no. 8).

#### 3.1.3 Knowing versus not knowing

In their retrospective accounts, participants recalled varying emotional responses to being aware of the aneurysm. While several described an initial period of fear, most reflected that these feelings did not persist or develop into debilitating anxiety. Concerns were generally described as situational rather than constant, typically emerging around follow-up appointments or at moments when the possibility of rupture felt more salient:

*I was scared at first - I was afraid for three years, before every examination. (…)*. *I thought about a brain hemorrhage, or bleeding (…)*. *But it’s not something I go around worrying about, it isn’t. It was mainly during the period when I was undergoing follow-up. (…)* (Participant no. 5).

A range of cognitive and emotional strategies for regulating concerns were described, including normalizing, focusing on the present, and downplaying risks. Some emphasized how reassurance from clinicians enabled them to bracket the issue and move on:

*“The feedback was that everything looked good, but they had found a sort of bulge in a blood vessel in the brain. Then, as I remember it, they said that they don’t treat this, it doesn’t require treatment. And that’s what I remember about it, and then I had forgotten about it.”* (Participant no. 7).

A recurring distinction was between knowing and worrying. For many, increased awareness of the aneurysm did not amplify anxiety but instead provided a sense of orientation and a framework for interpreting bodily sensations:

*“I do think it was a good thing that they found it. I really do, because otherwise I would have lived in complete uncertainty. Now I know that if I start to get headaches, I will seek help. And that’s because I’m aware of the aneurysm and what they told me - that it could be a cause*.*”* (Participant no. 9).

For others, this new knowledge introduced new uncertainties, particularly when they felt that the clinical implications were ambiguous and no intervention was indicated:

*“Initially, it was described as fairly large - roughly medium-sized. However, the more we talked, the smaller it appeared to become (…)*. *It was not regarded as a major risk, and as the doctor grew more irritated, the size seemed to diminish.”* (Participant no. 1).

The degree of concern was closely tied to perceived control and actionability. When participants felt there were concrete steps they could take, like monitoring symptoms, attending follow-ups and adjusting lifestyle, they described that worry was more manageable. In contrast, when the finding was perceived as non-actionable, uncertainty could lead to frustration or rumination.

Ambivalence emerged when participants were asked whether they would have preferred not to know about the aneurysm. Reflections unveiled a tension between the burden of carrying unactionable diagnostic knowledge and the hypothetical value of knowing whether treatment had been possible:

*“I didn’t need to know about the aneurysm, given that it is as it is now and nothing can be done about it. (…) On the other hand, if it was possible to do something, like an operation, then it would have been better to have the examination - if it could have prolonged my life. (…) it might stop the person from having a brain hemorrhage and ending up like a ‘vegetable,’ I mean, getting seriously damaged in the head (…).”* (Participant no. 5).

### 3.2 Maintaining normality and identity

#### 3.2.1 Maintaining everyday life

Participants recalled how maintaining their everyday life had involved meaning-making, emotional reframing, and drawing on personal histories to sustain a sense of wellbeing.

For some, a form of resilience was described as a part of their identity, enabling them to balance vulnerability with a commitment to living well:

*“If something bothers me, I’m able to adapt (…)*. *I think my family sees me as a cheerful person.”* (Participant no. 8).

Others framed their approach through narratives of survival and perspective, using earlier challenges as a reference point:

*“I had a very difficult upbringing. (…) I chose to see myself as a survivor, and I have lived according to that conviction ever since.”* (Participant 1).

These narratives highlight a dynamic and multifaceted process, involving both internal strategies such as acceptance, reframing and focusing on positive experiences, and external expressions of identity such as maintaining social engagement and a positive self-image. Participants who described themselves as emotionally stable, positive or relaxed often linked these traits to their ability to manage uncertainty:

*“I think I’m a person who has a very good mood and gets along with most people. (…) I don’t make a big deal out of things or worry much. I have a relaxed attitude toward life.”* (Participant no. 10).

Encounters with illness or loss among close others also heightened awareness of life’s fragility. Yet participants often described actively regulating these thoughts to prevent them from becoming overwhelming:

*“It does make an impression when people close to you are gone. Some of my friends got ill, and it all happened so quickly. That probably influenced that brief thought - “could it actually be serious after all?” But then I told myself I can’t start changing my perspective now.”* (Participant no. 7).

#### 3.2.2 Being healthy while having a diagnosis

Participants recalled how they negotiated the meaning of the diagnosis in relation to their broader sense of identity. Across interviews, they emphasized that the diagnosis influenced their awareness of health but did not fundamentally alter their self-image as generally healthy or capable individuals.

A recurring pattern was the use of adaptive self-presentation, where participants balanced acknowledgement of vulnerability with a desire to maintain normality and avoid burdening others. This often involved downplaying personal needs or distress, reflecting both self-protection and sensitivity to social expectations:

*“I am very adaptable and tend to fit easily into different environments. I think I am generally seen as quite easygoing - perhaps even too easygoing - in that I do not assert my own needs sufficiently.”* (Participant no. 4).

Participants frequently distinguished between having a diagnosis and being ill, underscoring the importance of preserving a healthy self-concept. Concerns about how others might interpret their condition sometimes led to concealing medication use or symptoms, revealing an underlying tension between outward confidence and internal vulnerability:

*“I don’t dare show people how much medication I take - then they’d think I’m ill, ha-ha. I do not see myself as ill.”* (Participant no. 8).

Comparisons with peers facing more severe health problems also served as a strategy for maintaining positive self-perception. This reinforced a sense of relative wellbeing:

*“When it comes to my health, I can’t complain. Compared to my peers who really have problems, I’m doing well.”* (Participant no. 9).

### 3.3 Translating the diagnosis into action and acceptance

#### 3.3.1 Disclosure, family and social consequences

Participants’ retrospective accounts revealed that living with a non-occluded UIA shaped social interactions, communication patterns, and strategies for managing how others perceived them. Many described navigating a tension between privacy, selective disclosure, and the desire to avoid being viewed as vulnerable or eliciting unnecessary concern.

A recurring pattern was the use of withdrawal or controlled openness as coping strategies. Some preferred to keep the diagnosis private in everyday settings, yet disclosed it when directly asked, balancing emotional expression with a need for social containment:

“*I tended to withdraw at work (…). But when someone asked, I would tell everything, including that I was afraid. My way of coping is simply to withdraw and become quiet.”* (Participant no. 5).

Family dynamics also played a significant role. The diagnosis raised questions about genetic susceptibility, prompting relatives to undergo screening. This extended the psychosocial impact beyond the individual, creating a sense of shared vulnerability which shifted the concern from a personal to a broader family issue:

*“My brothers also got checked (…). They said that my siblings should be screened, so both have undergone brain scans.”* (Participant no. 5).

#### 3.3.2 Lifestyle and health behavior

Many participants recalled that receiving the diagnosis prompted them to consider lifestyle adjustments, ranging from increased attention to health behaviors to minimal or no change.

Many described a heightened awareness of general health, even when they felt unable to influence the aneurysm directly. Maintaining physical activity, reducing stress, and adopting healthier routines were described as meaningful ways of preserving agency in a situation characterized by limited control:

*“With this condition in my head, there is nothing one can really do or influence. So, the only option is to live reasonably healthily and keep physically active.”* (Participant no. 1).

Lifestyle changes were also shaped by cardiovascular risk factors identified during follow-up. In these cases, the UIA catalyzed broader health awareness without being the primary driver of change:

*“It has not been related to the aneurysm. Rather, the detection of elevated blood pressure and cholesterol levels might have led to recommendations for modifications to diet and lifestyle, which I have followed.”* (Participant no. 2).

While not all participants made explicit lifestyle changes, many used the information as an opportunity to reassess health priorities, reinforcing a sense of agency and supporting long-term wellbeing. For some, the diagnosis contributed to long-term shifts in self-care and work-related behavior, encouraging greater attention to physical strain and overall health maintenance. These adjustments were framed as protective and future-oriented, supporting both wellbeing and continued participation in daily life:

*“Maybe I would have had a shorter remaining working life if I had not found out about the aneurysm. Because now I have also lowered my blood pressure and taken better care of myself, by avoiding extreme physical strain.”* (Participant no. 8).

#### 3.3.3 Agency, acceptance and the limits of action

Participants’ retrospective accounts also indicated that adherence to medication or lifestyle modification was not solely driven by clinical recommendations; it was also influenced by broader reflections on mortality, autonomy and the meaning of life.

For some, the diagnosis prompted philosophical responses. These participants described limited anxiety and framed mortality as an inevitable aspect of life, suggesting that existential acceptance rather than distress guided their health behaviors:

*“I rarely feel anxious (…). For me, the way I see it is that life eventually comes to an end regardless of how one lives it.”* (Participant no. 2).

Others acknowledged the potential severity of rupture, yet situated these thoughts within a realistic understanding of risk, balancing awareness with emotional steadiness:

*“I was a bit concerned … I mean, if that were to rupture, it would lead to a brain hemorrhage (…)*. *Then you would either be seriously or permanently harmed, if you didn’t die, I think.”* (Participant no. 4).

Participants frequently integrated the diagnosis into broader considerations about how they wanted to live, weighing the value of interventions against personal priorities such as independence, dignity and quality of life. These reflections shaped their willingness to adhere to recommendations and their views on the limits of medical intervention:

*“If I reach 75 and it ruptures, then it ruptures (…)*. *Being bedridden and tied to a hospital bed - that's something I’m not interested in.”* (Participant no. 8).

## 4 Discussion

### 4.1 Key results

This study captured retrospective narratives of experiences among 10 participants who were diagnosed with a UIA but not treated with occlusion of the aneurysm. The main findings were that many recalled experiencing serious worry, and in some cases fear, shortly after the diagnosis. Most described a subsequent process of adaption to the uncertainty through trust in clinicians, resilience, normalization, and meaning-making, which enabled them to reestablish normality in everyday life. Concerns were generally described as diminishing over time, but it could resurface around follow-up imaging. Few participants described persistent anxiety at the time of the interview, a median of eight years after diagnosis. The diagnosis increased attention to health behaviours, and motivated healthy lifestyle changes for many participants.

### 4.2 Strengths and limitations

It is a strength that we interviewed a subsample of a well-characterized population-based cohort which we have studied quantitatively previously (Rosenlund et al., 2024). However, studies indicate that health survey participants have higher socioeconomic status and better health than non-responders (Jacobsen et al., 2012; Eggen et al., 2013). This can cause selection bias towards recruitment of participants with better than average ability to utilize counseling and develop coping strategies.

The timing of the interviews several years after the diagnosis provides insights into participants’ retrospective accounts of longer-term experiences, a perspective rarely represented in the literature. It is, however, a weakness that the cross-sectional design with retrospective reporting of narratives rather than prospective observation of trajectories entails a risk for recall bias. A prospective design with serial interviews would have produced more reliable data, since recollections may have been influenced by subsequent experiences, comorbidity and ageing. The cross-sectional design can also cause survivorship bias because participants in the cohort we recruited from who died early after diagnosis may have had other experiences than those who lived on and were eligible for inclusion. In addition, we considered data saturation achieved after 10 interviews but cannot exclude the possibility that the six additional individuals who had consented could have enriched the data.

It is a strength that the interviews were conducted by an investigator who had not been involved in the participants’ medical care and had no information about their health except the diagnosis. However, the interviews were conducted in the neurosurgical outpatient clinic where participants had received follow-up care. This implies a risk for power imbalance and social desirability bias towards more positive expression of the experiences.

We intended to not hold specific assumptions about possible findings and used reflexivity and investigator triangulation including different professional perspectives to mitigate interpretation bias. We cannot, however, exclude that subconscious assumptions influenced our analyses.

It also is a limitation that we studied participants managed by a few neurosurgeons at one department. Communication practices vary, and other clinicians or healthcare systems may have produce different experiences. Moreover, the neurosurgeons involved in the follow-up of these health survey participants were ethically obliged to avoid unnecessary worry after the survey. This limits generalizability to patients diagnosed with incidental UIAs in regular clinical settings. Additionally, for ethical reasons, we did not explicitly probe concerns about heredity, screening of participants’ children, or restraints on sexual life during the interviews. This may have restricted the width of captured themes.

### 4.3 Interpretation

Qualitative studies are not intended to produce generalizable findings. However, despite the limitations, the present study provided in-depth insight into participants’ immediate and long-term experiences and perspectives that would not have been captured through questionnaire-based methods. To our knowledge, Jelen and co-workers published the only previous similar study (Jelen et al., 2023). They did semi-structured telephone interviews median 14 months after the UIA diagnosis and reported that reactions such as anxiety, sadness and worry had entailed lifestyle changes including e.g. avoidance of straining-intensive exercise and modification of work-related performance (Jelen et al., 2023).

The present study mainly confirms this pattern, as participants recalled a similar burden when first informed about the aneurysm. However, our findings indicate a temporal trajectory in which distress diminished over time. This is consistent with Penchev and co-workers’ review and meta-analysis of previous quantitative evidence (Penchev et al., 2025). Taken together, the available qualitative and quantitative evidence suggest that while UIA detection can provoke substantial initial worry, many individuals adapt over time, particularly when communication is clear and follow-up is structured.

Importantly, we found a wide spectrum of responses and temporal patterns, ranging from minimal impact to more severe and persistent distress, suggesting substantial heterogeneity of psychosocial outcomes (Rosenlund et al., 2024). We assume that subtle but important reactions are unlikely to register in cross-sectional quantitative studies using standardized instruments and suggest that use of qualitative methods is necessary to reveal psychosocial burdens that may coexist with normal scores on generic quantitative measures of anxiety, distress, and quality of life.

The findings should also be interpreted in the context of the structured setting and follow-up in the Tromsø7 study. In this population-based health survey, participants might have had less reason to worry than in clinical settings: they were asymptomatic individuals who voluntarily underwent brain MRI, and thus probably had good baseline health and low pre-existing worry. Nevertheless, we found that disclosure of a UIA could cause concern even under such optimal conditions. We therefore suggest that patients should be informed about the possible burden of incidental findings in clinical settings, e.g. when they request referral to MRI on weak indications or before screening of individuals with a family history of UIA.

## 5 Conclusion

In this qualitative study of selected health survey participants who were diagnosed with a UIA without undergoing treatment with occlusion of the aneurysm, many retrospectively recalled experiencing serious worry and, in some cases, fear shortly after the diagnosis. Most participants described having adapted and developed ways of coping over time, although concerns could resurface around follow-up imaging. We suggest these insights could inform decision-making in contexts where clinicians and patients must balance the potential benefits and burdens of imaging and surveillance.

## Ethics declaration

Written informed consent to take part in the study and to publish the article has been obtained from all participants or their legal representatives. The privacy rights of participants have been observed.

This study was performed in compliance with relevant laws, regulatory frameworks and guidelines where the research took place. This study was approved by the REK north (Approval No. 126174/2020).

## Data availability statement

The data are not available due to their sensitive nature.

## Patient involvement

A representative from the Norwegian Heart and Lung Patient Organization (LHL) Young Patients’ Stroke Group – the Norwegian organization for young stroke survivors was involved in reviewing the manuscript and provided structured feedback based on user and patient-organization experience.

## Declaration of generative AI and AI-assisted technologies in the writing process

During the preparation of this work, the authors used Microsoft Copilot to improve language quality. After using this tool/service, the authors reviewed and edited the content as needed and take full responsibility for the content of the published article.

## Funding

Dr Vea Iversen has received a research fellowship from the UiT the Arctic University of Norway and a research grant from the Odd Berg Group Research Fund.

## Declaration of competing interest

The authors declare that they have no known competing financial interests or personal relationships that could have appeared to influence the work reported in this paper.
