# Supplementary material for: Experiences of being diagnosed with unruptured intracranial aneurysm in a population-based health survey: A qualitative interview study
Source: Brain Spine. 2026 Oct 9;6:107469. doi: 10.1016/j.bas.2026.107469 (PMC13679309; doi:10.1016/j.bas.2026.107469)
Supplement: Supplementary file 1 [file mmc1.docx]

| **Theme** | **Main questions** | **Probing questions** |
| --- | --- | --- |
| Introduction | - Welcome - Information about the interview. Introduction of the interviewer |  |
| Background and participant | - Could you tell a little about yourself? | - Personal information (name, date of birth) - Age, place of residence, family, job, hobbies, health status, etc. |
|  | - How would you describe yourself as a person? | - How would your friends or family describe you? - What do you enjoy doing in your everyday life? - Do you tend to worry easily about things, or not? - How do you handle challenges? - Ability to accept and adapt vs getting stuck in problems; positive vs negative attitudes toward uncertainty and challenges |
|  | - How is your everyday life? |  |
|  | - Do you have any previous illnesses, diagnoses, or health problems? | - Do you use any medications? |
| Description of situation at the time of diagnosis | - When did you participate in the Tromsø Study? | - Why did you participate? Can you tell me about what they did there? Did they find anything? |
|  | - How would you describe your health prior to participating in the Tromsø Study? |  |
| Experiences and reflections after the diagnosis | - As you could read in the letter, I would like to talk with you because you have been diagnosed with an intracranial aneurysm. But could you start by explaining to me how you received the diagnosis, and what your first thoughts were? | - How did it affect you? - How did you react when you were told? - Did you feel afraid? If yes, what were you afraid of?   - Possible concerns: bleeding and death; bleeding, treatment and survival with severe disability; the need for future elective treatment; being a burden to the family? |
|  | - Can you tell me about your understanding of what an aneurysm — a bulge in a blood vessel in the brain — is |  |
|  | - Had you heard about intracranial aneurysms before? | - Yes → What did you know, and how did you know it - No → Have you received any information about the diagnosis? From whom? What were you told - What do you know about the importance of size, location, etc |
|  | - Have you told anyone about the diagnosis? | - If yes – whom did you tell, and how did they react - Do others in your family or relatives have the same diagnosis |
|  | - Do you feel that you have changed since you learned about the diagnosis? Has it changed the way you see yourself? - Has it influenced how you think about your own health? | - Sick or healthy - Is there anything you need to pay particular attention to after receiving the diagnosis? For example, do you think you have an increased risk of anything? What and how? - Some people experience fear, worry, or guilt when they receive such a diagnosis. Is that something you recognize? What, why, and how? - Others may feel relief because they then know more about their own health. Is that something you recognize? If yes, in what way - Do you think more about the future after receiving the diagnosis? - Do you feel that you have become more or less worried (about your health) after learning that you have an aneurysm |
|  | - Is there anything you do differently after receiving the diagnosis? | - For example, this could include eating differently, changing your physical activity, or thinking more about family relationships |
|  | - Do you feel that you should have had more or less follow‑up or treatment? | - Is there any follow‑up or treatment you would like to have, but that the doctors believe you do not need? - If we imagine that you had never been told you have an aneurysm — what would that mean for you? - ONLY if worried: What if you had not received the diagnosis — would you then not have been worried? |
|  | - Could you tell me a bit more about your everyday life? | - What do you do during the day? Have you always enjoyed doing these things? |
| Living with an aneurysm | - I would now like to talk with you about the period several years after the aneurysm was detected - How would you describe the time after you learned that you have an aneurysm? | - Doctor appointments with your GP or at the hospital? - Are you working? On sick leave?   - If yes: why were you on sick leave, and for how long? |
|  | - Is your life affected by the information you received? | - Lifestyle habits - Joy of life - Mental health - Routine question (camouflage): Has it affected your sexuality?   - Afraid of rupture? |
|  | - What feelings do you associate with the diagnosis? | - Anger, shame, guilt? - Do you feel sick? |
|  | - If you could turn back time, would you have preferred not to know about the aneurysm? | - Why/why not? |
| Other | - Is there anything else you think we should have talked about in this context that we have not covered? |  |
| Closing questions | - How did you experience being interviewed |  |
