# Supplementary material for: Experiences of being diagnosed with unruptured intracranial aneurysm in a population-based health survey: A qualitative interview study
Source: Brain Spine. 2026 Oct 9;6:107469. doi: 10.1016/j.bas.2026.107469 (PMC13679309; doi:10.1016/j.bas.2026.107469)
Supplement: Supplementary file 2 [file mmc2.docx]

Supplementary Table 1. Distribution of illustrative quotations across participants and main themes.

| Main theme | Participants represented by quotations |
| --- | --- |
| Receiving and making sense of uncertain knowledge | P1, P5, P6, P7, P8, P9 |
| Maintaining normality and identity | P1, P4, P7, P8, P9, P10 |
| Translating the diagnosis into action and acceptance | P1, P2, P4, P5, P8 |
